# p21-activated kinase 1: PAK'ed with potential

**DOI:** 10.18632/oncotarget.271

**Published:** 2011-06-07

**Authors:** Christy C. Ong, Adrian M. Jubb, Wei Zhou, Peter M. Haverty, Adrian L. Harris, Marcia Belvin, Lori S. Friedman, Hartmut Koeppen, Klaus P. Hoeflich

**Affiliations:** ^1^ Department of Translational Oncology, Genentech, Inc., South San Francisco, CA 94080, USA; ^2^ The Weatherall Institute of Molecular Medicine, University of Oxford, Headington, Oxford OX3 9DS, UK; ^3^ Department of Bioinformatics, Genentech, Inc., South San Francisco, CA 94080, USA; ^4^ Department of Pathology, Genentech, Inc., South San Francisco, CA 94080, USA

**Keywords:** PAK1, apoptosis, squamous, lung cancer, breast cancer

## Abstract

The p21-activated kinases (PAKs) are central players in growth factor signaling networks and morphogenetic processes that control proliferation, cell polarity, invasion and actin cytoskeleton organization. This raises the possibility that interfering with PAK activity may produce significant anti-tumor activity. In this perspective, we summarize recent data concerning the contribution of the PAK family member, PAK1, in growth factor signaling and tumorigenesis. We further discuss mechanisms by which inhibition of PAK1 can arrest tumor growth and promote cell apoptosis, and the types of cancers in which PAK1 inhibition may hold promise.

## PAK1 SIGNALING IN NSCLC

**Figure 1:**
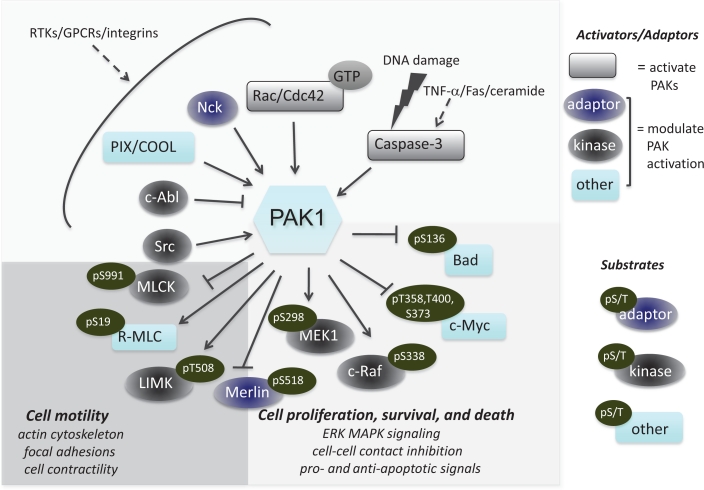
PAK1 signaling pathways in mammalian cells Upstream activators of PAK1 include receptor tyrosine kinases (such as Met, HER3 and PDGFR), integrins and G-protein coupled receptors. Well-established effectors regulating cellular motility, proliferation and survival are shown. PAK1 acts on a number of regulators of the cytoskeleton, including to increase actin and tubulin polymerization.

PAK serine/threonine protein kinases are subdivided into two groups, PAK1-3 (group I) and PAK4-6 (group II), based on sequence similarities and an autoinhibitory domain which is present in group I, but not group II PAK proteins [1,2]. The group I PAKs share a number of other conserved structural characteristics, such as a p21-binding domain (PBD), a serine/threonine kinase domain, an acidic region and multiple proline-rich regions that serve as binding sites for SH3 domain-containing proteins. As a major downstream effector of the Rho family small GTPases Cdc42 and Rac1, PAK1 plays a fundamental role in controlling cell motility by linking a variety of extracellular signals to changes in actin cytoskeleton organization, cell shape and adhesion dynamics (Figure 1) [3,4,5].

PAK1 is the most well characterized family member and is widely expressed in a variety of normal tissues [1]. PAK1 expression is significantly increased in ovarian, breast and bladder cancers [6,7,8]. In luminal breast cancer, the expression and localization of PAK1 protein was recently assessed in primary tumors from 403 premenopausal patients that were randomized to two years of adjuvant tamoxifen or no treatment [9]. Elevated expression and/or nuclear localization of PAK1 were associated with resistance to tamoxifen therapy [9], possibly occurring as a result of direct phosphorylation and ligand-independent transactivation of estrogen receptor-α by PAK1 [10]. Functional studies have also implicated PAK1 in cell transformation [11], and transgenic overexpression of PAK1 in the mammary gland promotes the formation of tumors and preneoplastic lesions in animal models, albeit with a long latency [12]. These findings indicate that PAK1 may contribute to tumorigenesis in some disease contexts [13,14].

To determine the possible extent of PAK1 dysregulation across human cancers, we determined PAK1 protein expression and subcellular localization via immunohistochemical (IHC) staining of primary human tissue microarrays from several tumor indications, including breast, lung, and head and neck cancers [15]. Nearly two-thirds of squamous NSCLC samples in our panel were positive for PAK1 protein expression and 52% (n=67) of all cases showed staining of moderate or strong intensity in the malignant cells. In comparison, adjacent normal lung tissue did not express appreciable protein levels of PAK1. Nuclear localization of PAK1 was also evident in a significant proportion of squamous NSCLC tumors (25%) and it has been shown that nuclear import of PAK1 can play a critical role in vertebrate cell biology [16]. Supporting evidence that PAK1 expression is elevated in squamous NSCLC was also obtained by analyzing PAK1 mRNA expression in a distinct set of laser-capture microdissected lung tissues (p < 0.0001).

**Figure 2:**
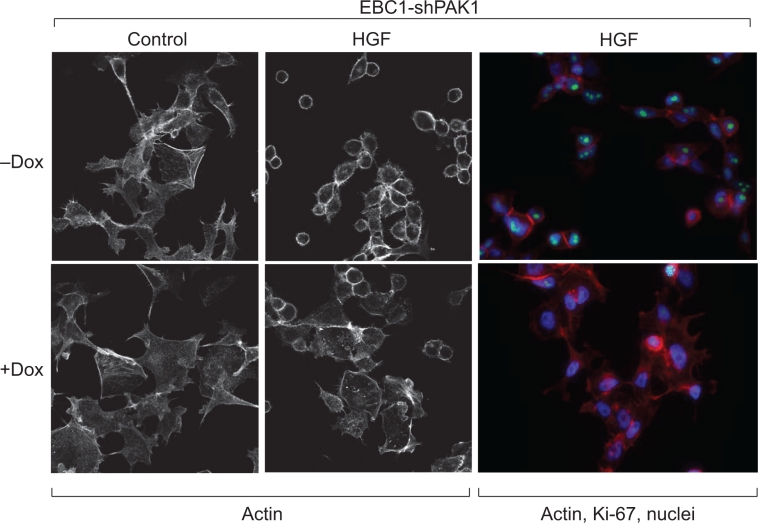
PAK1 regulates actin cytoskeleton morphology and proliferation of squamous NSCLC cells PAK1 is required for hepatocyte growth factor (HGF)-induced actin dynamics and proliferation of squamous NSCLC cells. EBC-1 cells were pretreated with Dox for 3 days prior to serum starvation for 16 hours and treatment with 50 ng/mL HGF for 1 hour. Cytoskeleton morphology and cell cycle progression were detected by staining with phalloidin and Ki-67, respectively. Inducible-knockdown of PAK1 attenuated cell rounding and cortical actin distribution, as quantified by measuring cell diameter by image analysis, and reduced nuclear levels of Ki-67. Induction of control LacZ shRNA with Dox had no effect in these assays.

We subsequently examined the effect of RNAi-mediated knockdown of PAK1 in a panel of lung cancer cell lines to clarify the contribution of PAK1 towards tumor cell growth and survival. Transient knockdown of PAK1 and PAK2 together resulted in a 2.5- to 8-fold reduction in [^3^H]-thymidine incorporation of multiple squamous NSCLC lines when compared with control cells (p<0.0001). In addition, we made use of a doxycycline-inducible short-hairpin RNA (shRNA) system [17,18] to study PAK1 loss-of-function effects. Tightly regulated Dox-mediated knockdown of PAK1 was observed in NSCLC cells and tumor xenografts. Inhibition of PAK1 resulted in accumulation of cells in the G_1_ phase of the cell cycle, altered levels of E2F and p27^Kip1^ (which play multifaceted roles in regulation of G_0_ to S phase transitions of the cell cycle), and inhibition of *in vivo* tumor growth [19,20]. Furthermore, PAK1 knockdown substantially decreased tumor cell proliferation (imaged by immunofluorescence staining of Ki-67 positive nuclei), migration and actin dynamics induced by hepatocyte growth factor (HGF) treatment (Figure 2). Amplification or over-expression of c-MET (the receptor for HGF) is a known genetic aberration and therapeutic target in squamous NSCLC [21,22] and PAK1 may be a key effector for HGF/c-MET signaling in cancer. Together, these findings demonstrate that PAK1 is important for proliferation of a subset of squamous lung cancers *in vitro and in vivo*, and support the possibility that interfering with PAK1 activity could have therapeutic efficacy in this indication.

## IMPLICATIONS FOR TREATMENT OF SQUAMOUS LUNG CARCINOMA

Primary lung cancer is a heterogeneous disease both genetically and histologically. It can be subdivided into small cell lung cancer (accounting for approximately 20%) [23,24], which has a neuroendocrine phenotype, and non-small cell lung cancer, which comprises adenocarcinomas, squamous carcinomas and more rare subtypes. Squamous lung carcinoma is a prevalent subtype, accounting for 33% of primary lung cancers in non-smokers and 42% in smokers [23,24]. Historically, differences in the prognosis and sensitivity to chemotherapy of adenocarcinomas and squamous carcinomas (following correction for stage and other confounding variables) were considered insufficient to warrant different treatment. First line treatment with one of four combination chemotherapy regimens was once the standard of care, irrespective of a tumour’s histology or genetic makeup, but despite therapy two-year survival is only 11% [25]. However, since that time several targeted therapies have been approved for NSCLC. Bevacizumab, an antibody to VEGF that targets tumour blood vessels, has shown improvements in overall and/or progression-free survival when combined with cytotoxic chemotherapy [26,27]. However, the use of bevacizumab specifically excludes squamous NSCLC due to haemoptysis [28]. Erlotinib is a small molecular tyrosine kinase inhibitor against epidermal growth factor receptor (EGFR) that is approved as monotherapy for maintenance treatment following platinum-based chemotherapy or as monotherapy following the failure of ≥1 chemotherapy regimen [29]. While erlotinib is approved for use in unselected patients, there is evidence that erlotinib and a related drug, gefitinib, show greater activity and efficacy in cancers that harbour specific mutations in EGFR and are wildtype for KRAS, an oncogene that transduces growth promoting signals downstream of EGFR [30]. EGFR mutations are very frequent in lung adenocarcinomas (40%), but are infrequent (0-5%) in squamous cell carcinomas of the lung [31,32]. Moreover, other mutations affecting genes that impinge on the EGFR/RAS pathway are relatively infrequent (3% PI3KCA, 6% KRAS, 2% BRAF and 10% PTEN) [31]. Thus, squamous lung carcinoma represents an unmet medical need with a poor prognosis and PAK inhibitors could be tested in this indication.

## PAK1 SIGNALING AND BREAST CANCER

PAK1 genomic copy number and protein expression level were also ascertained in 216 and 274 human breast carcinoma samples, respectively [15]. PAK1 expression was absent in normal breast epithelial cells, but genomic amplification was prevalent in luminal subtype tumors and elevated cytoplasmic protein expression was observed across 39% of primary adenocarcinomas. Consistent with the evolutionarily conserved role for PAK1 in regulating cell motility, high PAK1 expression was associated with lymph node invasion and occurred more frequently in nodal metastases compared to primary tumors. It is interesting that the frequency of dysregulated expression of PAK1 was more frequent than would be predicted by genomic amplification alone and additional regulatory mechanisms that may increase PAK1 expression in breast cancer, such as via microRNA genes [33], remain to be fully explored. Analysis of MDA-MB-175, HCC1500 and MDA-MB-134IV cell lines with PAK1 genomic copy number gain revealed exquisite dependence on PAK1 expression and activity for cell survival [15]. The pro-survival function of PAK1 in breast cancers might be another contributing factor to the association of elevated PAK1 expression and reduced clinical benefit in patients treated with tamoxifen [9]. Taken together, our identification of PAK1 as an “Achilles’ heel” for a subpopulation of breast cancer provides evidence of oncogene addiction [34] and a rationale for PAK1-directed therapy in this disease indication.

## RATIONAL THERAPEUTIC STRATEGIES FOR COMBINING WITH PAK1 INHIBITION

Despite the exquisite sensitivity of the breast cancer cell lines harboring genomic copy gains to PAK1 knockdown, inhibition of PAK1 did not generally increase apoptosis of NSCLC cells and xenograft models. We therefore hypothesized that PAK1 inhibition may synergize with other molecularly targeted agents to augment killing of tumor cells. We also reasoned that testing the combination of PAK1 inhibition plus compounds of known cellular mechanism would aid in understanding the cellular function of PAK1 in NSCLC cells as well as in rationally designing effective combination regimens that could be readily translated into the clinic. Hence, a cellular viability screen was performed using shPAK1 isogenic cells and a panel of 200 small molecule compounds that included oncology drugs approved by the FDA, signaling pathway inhibitors and DNA damaging agents. Among the tested compounds, antagonists of inhibitor of apoptosis proteins (IAP), epidermal growth factor receptor (EGFR), MAPK/ERK kinase-1/2 (MEK1/2) and Src family kinases displayed enhanced efficacy in the context of combined PAK1 knockdown [15]. A number of antagonists have been described to disrupt the association of IAP with the second mitochondrial activator of caspases (SMAC) and activated caspase-9 [35,36] and this protein family was selected for follow-up studies. Consistent with the small molecule screening data, strong combinatorial activity and induction of programmed cell death were confirmed for PAK1 and IAP dual inhibition with EBC-1 and additional NSCLC cell lines. Our recent published work demonstrates a fundamental role for PAK1 in NSCLC biology and provides support for PAK1 as a therapeutic target in this tumor indication.

## CONCLUSIONS

In summary, recent work on the PAK family of kinases has focused on the role of these proteins in cellular functions relevant to tumor initiation and maintenance, namely cell proliferation and survival signaling [13,37,38,39]. We assessed the role of PAK1 in a large panel of human tumors (via high resolution, single-nucleotide polymorphism arrays and immunohistochemical staining) and *in vivo* tumor models (via inducible RNA interference) and showed that PAK1 inhibition resulted in improved anti-tumor efficacy [15]. It will be of great interest to expand on these efficacy experiments by utilizing genetically engineered mouse models to continue evaluating the therapeutic benefit of PAK1 inhibition [40,41]. PAK1 inhibition also promoted tumor cell apoptosis as either single-agent treatment (in the context of tumor cells with focal genomic amplification of PAK1) or as combination therapy with several targeted agents (in squamous cell carcinomas). It will be important to more thoroughly characterize PAK1 effector signaling and possible molecular mechanisms for regulation of cell survival in squamous NSCLC. For instance, PAK1 signaling in squamous NSCLC cells was associated with an accumulation of the anti-apoptotic BCL2 family member, myeloid cell leukemia-1 (Mcl-1), and suggests a combination with BCL2 inhibitors such as Navitoclax/ABT-263 [42]. Nuclear factor κB (NF-κB) subunits were also differentially phosphorylated following PAK1 inhibition in multiple squamous NSCLC cell lines and this pathway may contribute to transformation of lung cancer cells [43,44]. Taken together, we describe evidence for dysregulation of PAK1 in breast and squamous NSCLC tumors and a role for PAK1 in cellular survival and proliferation in these indications.
